# Evolutionary patterns and research frontiers of macrophages in myocardial infarction: A bibliometric analysis

**DOI:** 10.1097/MD.0000000000043038

**Published:** 2025-06-27

**Authors:** Guo Yang, Jia Lin Zhong, Yang Xing, Chuan Wei Li, Hao Wang, Jun Xiao

**Affiliations:** a Chongqing Emergency Medical Center, Chongqing University Central Hospital, School of Medicine, Chongqing University, Chongqing, China; b Department of Clinical Laboratory, Chongqing Emergency Medical Center, Chongqing University Central Hospital, Chongqing, China; c Department of Cardiovascular Medicine, Chongqing Emergency Medical Center, Chongqing University Central Hospital, Chongqing, China.

**Keywords:** bibliometrics, CiteSpace, macrophages, myocardial infarction, VOSviewer

## Abstract

**Objective:**

The objective of this study was to evaluate the role of macrophages in myocardial repair following myocardial infarction (MI) through a bibliometric analysis of post-MI macrophage research.

**Methods:**

A comprehensive dataset was compiled from the Web of Science Core Collection, including articles published up to December 30, 2023. The analysis involved co-authorship networks and keyword co-occurrence, with visualization facilitated by VOSviewer and key terms identified through CiteSpace.

**Results:**

The study encompassed 1342 publications, highlighting the United States, China, and Germany as leading contributors to the field. Circulation Research emerged as the most active journal in publishing related studies. The prevalent themes were inflammation and atherosclerosis, indicating their significance in macrophage involvement post-MI.

**Conclusion:**

This study provides a detailed understanding of macrophage involvement in MI, outlining significant countries, institutions, journals, and publications. It offers valuable insights into macrophage function in MI, contributing to the broader understanding of myocardial repair mechanisms.

## 1. Introduction

Myocardial infarction (MI), a critical medical emergency, ensues, while cessation of the blood slide precipitates irreversible necrosis of cardiac myocytes.^[1]^ The past several decades have witnessed a marked escalation in MI prevalence, positioning it as a fundamental cause of mortality global.^[2]^ In the MI paradigm, macrophages are critical immune elements orchestrating the inflammatory cascade and reparative procedures such as cardiac insult. Subsequent to MI, macrophages modulate the importance of myocardial damage and convalescence trajectory via the secretion of cytokines and protein modulation.^[3]^ Empirical evidence has revealed the ability of macrophages to transdifferentiate into diverse phenotypes, undertaking roles within the phagocytosis of dying cells, neovascularization, fibrogenesis, and scar tissue maturation, each vital to cardiac practical restitution.^[4]^ Regardless of the proliferation of macrophage-centric studies in MI research, there is a deficit of methodical syntheses. An exigency for comprehensive bibliometric scrutiny of scholarly outputs, geopolitical contributions, institutional involvement, magazine media, authorship, and thematic keywords persists. Bibliometrics, the subject that harnesses quantitative and statistical methodologies to dissect the genesis and propagation of literature, assumes an essential function in the medical sphere. It provides insights into the ramifications and dissemination of clinical inquiries.^[5]^

This study aims to fill this methodological void by conducting a bibliometric analysis of the macrophage-centered MI study landscape. By leveraging the electricity of bibliometrics, we are searching to explain the key research trends, perceive the most impactful studies, and highlight the collaborative efforts among researchers worldwide. Our evaluation contains a holistic view of the literature, along with the identification of know-how clusters and visualization of co-citation and co-authorship networks. This method will not best map the cutting-edge country of macrophage studies in MI, but also reveal capability regions for future research and interdisciplinary collaboration.

## 2. Materials and methods

### 2.1. Data source and literature search strategy

The Web of Science (WoS) was selected as the primary database for this research because of its exhaustive coverage of more than 12,000 scholarly journals and frequent use by researchers. Compared to other databases, such as Scopus, Medline, and PubMed, WoS offers the most comprehensive and reliable bibliometric analysis. On December 30, 2023, a search was performed across all available database editions, and pertinent articles were exported from the WoS Core Collection (WoSCC). The search strategy was unanimously agreed upon by all authors following consultation with a senior literature search expert, adhering to the following criteria: TS = (“acute myocardial infarction”) OR TS=(“myocardial infarct”) OR TS=(“heart attack”) OR TS=(“Cardiovascular Strokes”)) AND (TS=(“macrophage”) OR TS=(“macrophages”)).

To facilitate the subsequent analysis of the literature, the types of articles considered were limited to Articles and Review Articles, and the language was restricted to English. Complete records and references cited in relevant publications were extracted, saved in plain text format, and stored as download_*. txt files for further study.

### 2.2. Software for bibliometric analysis

This study utilized R version 4.3.3,^[6]^ VOSviewer,^[7]^ and CiteSpace^[8]^ as the primary software tools for conducting bibliometric analyses. The Bibliometrix R package version 4.2.0^[9]^ was used to calculate the frequency of international collaboration among countries. VOSviewer was used to determine the number of publications, citation counts, and keyword frequency. Utilizing its embedded clustering algorithm, VOSviewer facilitates the construction and visualization of a co-occurrence network of key terms within the scientific literature. Co-authorship and co-occurrence analyses were central to this research, with these tools being instrumental in analyzing collaborations among nations, institutions, and authors. CiteSpace was used to identify highly cited references and keywords that exhibited a significant increase in citations over a specific period. Using the online bibliometric platform (https://bibliometric.com/), we visualized international collaborations between countries. The exponential growth function in Excel 12 was used to analyze trends in the publication numbers.

## 3. Results

### 3.1. Overview of publication status

**Figure 1.**
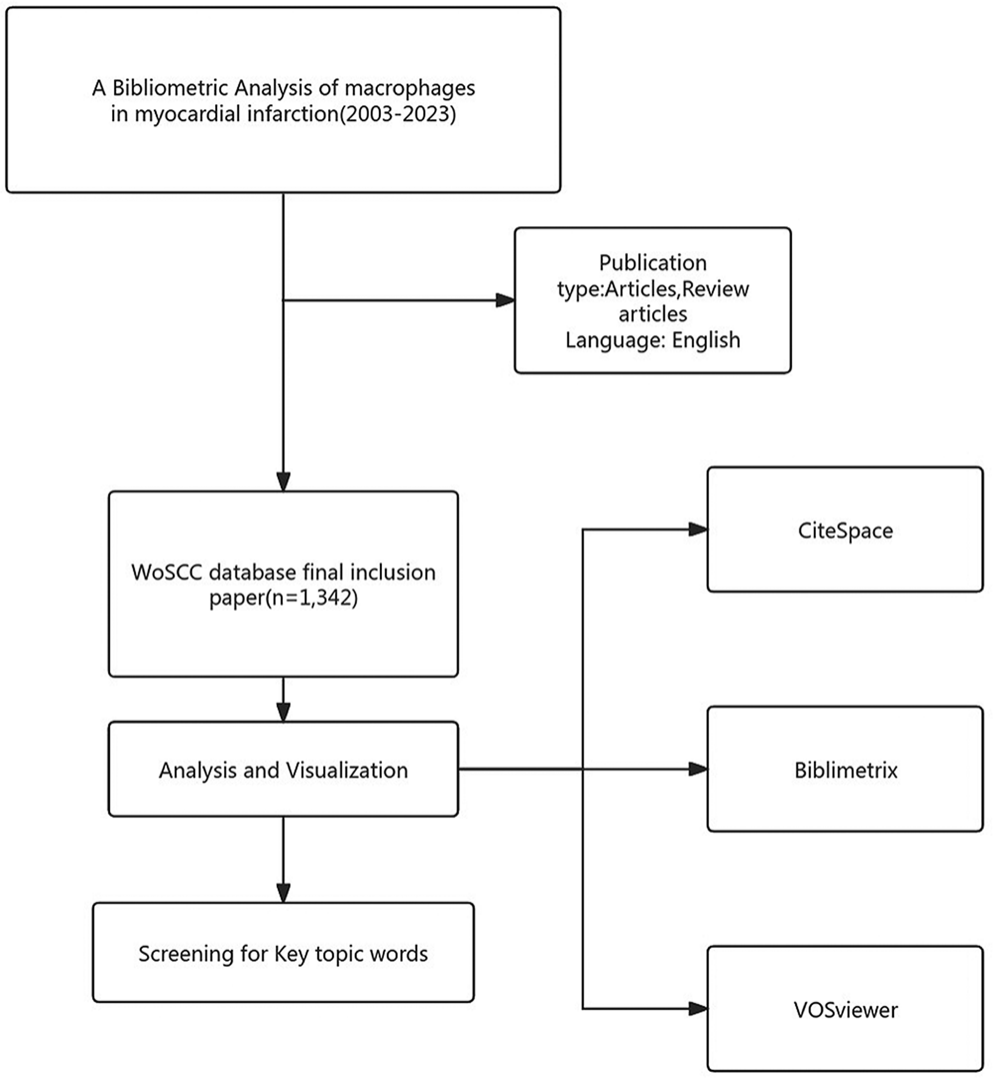
Flow-chart of the study.

**Figure 2.**
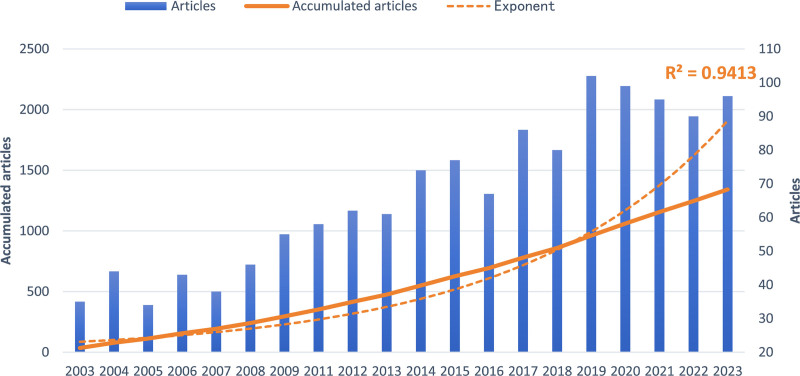
Number of publications per year and the cumulative number.

As illustrated in Figure 1, 1342 conventional articles pertaining to MI macrophages were included in this study. Figure 2 shows the annual and cumulative publication counts associated with MI macrophages. A steady increase was observed from 35 publications in 2003 to 96 publications in 2023, indicating a consistent increase in the cumulative numbers. An exponential growth function was then employed to assess the relationship between cumulative publications and publication years, aligned with the trend in cumulative publication numbers (*R*^2^ = 0.9184). This robust correlation signifies notable growth and evolution of MI macrophages.

### 3.2. Results of national publication counts

**Figure 3.**
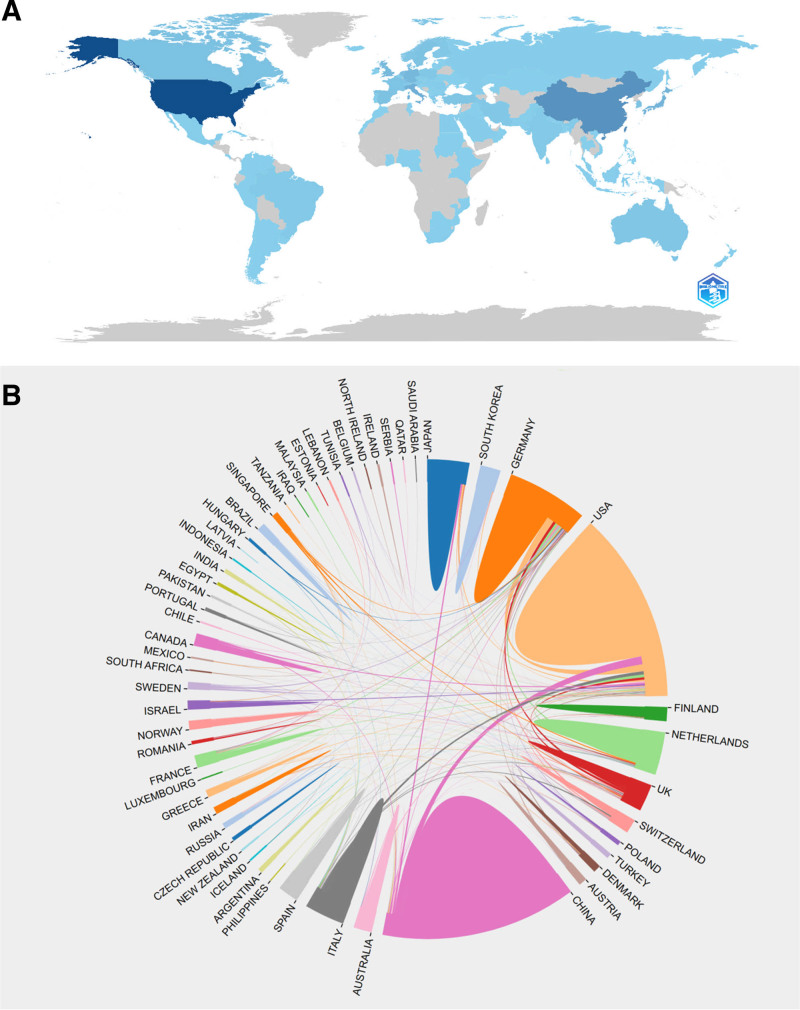
Each country’s contribution to the macrophages in myocardial infarction (A). Global collaborations of macrophages in myocardial infarction and the frequency of collaboration among nations (B).

An analysis of the volume of national publications was conducted to identify the leading contributors to this domain. As depicted in Table1 and Figure S1, Supplemental Digital Content, https://links.lww.com/MD/P271, the United States ranks first with a total of 427 publications, followed by China (330), Japan (153), Germany (149), and the Netherlands (82). As part of our survey, spatial distribution of research productivity was further visualized through geographical heatmaps in Figure 3A, and a collaborative network was constructed based on the number and relationship of publications in each country in Figure 3B. These findings indicate that the US is at the vanguard in the field of MI macrophage research, with China closely trailing. The most frequent domestic collaboration was observed in China. Internationally, the most prolific collaborators were the US and China (44), Germany (38), Italy (19), and the Netherlands (16), all of which were predominantly partnered with the US.

of the 34 countries that published 5 or more articles, we conducted a co-authorship analysis on the aforementioned 37 countries to investigate inter-country collaborations. In the cluster and temporal overlap networks, the size of circles represents the number of publications. The colors of the circles denote the intensity of collaboration between different countries within the cluster network. The color represents the average publication year of each country within a specific research domain in the temporal overlap network. As illustrated in Figure S2, Supplemental Digital Content, https://links.lww.com/MD/P271, the 37 countries formed 7 clusters. The red cluster encompasses the majority of the countries, including 10 countries. As shown in Figure S2, Supplemental Digital Content, https://links.lww.com/MD/P271, the US emerged as an early pioneer in the field of MI macrophages, while Chinese researchers have made more recent contributions in this area.

### 3.3. Results of national publication counts

To explore the contributions of institutions to MI macrophage research, an analysis of publication volume by various organizations was undertaken. Globally, approximately 1643 institutions are engaged in MI macrophage research. As shown in Table 1 and Figure S1, Supplemental Digital Content, https://links.lww.com/MD/P271, the top 20 institutions with the most publications in this field included 11 from the United States, 4 from China, 3 from the Netherlands, 2 from Germany, 1 from France, and 1 from the United Kingdom. Harvard University led 69 publications.

**Table 1:** Top 10 countries and institutions on the research of macrophages in myocardial infarction.

Rank	Countries			Institutions		
	Countries	Article counts	Percentage	Institutions	Article counts	Percentage
1	USA	427	31.818	HARVARD UNIVERSITY	69	5.142
2	Peoples R China	330	24.590	HARVARD MEDICAL SCHOOL	61	4.545
3	Japan	153	11.400	MASSACHUSETTS GENERAL HOSPITAL	53	3.949
4	Germany	149	11.102	UNIVERSITY OF CALIFORNIA SYSTEM	33	2.459
5	Netherlands	82	6.1102	UNIVERSITY OF AMSTERDAM	30	2.235
6	Italy	81	6.0357	BRIGHAM WOMEN S HOSPITAL	25	1.863
7	England	74	5.5141	INSTITUT NATIONAL DE LA SANTE ET DE LA RECHERCHE MEDICALE INSERM	24	1.788
8	Australia	42	3.1296	UNIVERSITY OF LONDON	24	1.788
9	Spain	42	3.1296	VRIJE UNIVERSITEIT AMSTERDAM	23	1.714
10	UK	40	2.9806	UNIVERSITY SYSTEM OF OHIO	22	1.639

To further investigate collaborations between institutions, a co-authorship analysis was performed on all publications. Figure S3, Supplemental Digital Content, https://links.lww.com/MD/P271 shows that 38 institutions published at least 10 papers each. These 38 institutions form 5 clusters, with the largest being the red cluster, comprising 14 institutions, predominantly from China. Several US research institutions spearheaded by Harvard University have made significant early contributions to the development of MI macrophages. In contrast, post-2020, multiple Chinese research institutions led by Fudan University have increasingly participated in research on MI macrophages.

### 3.4. Results of journals and co-cited academic journals

#### 3.4.1. Results of publication quantity and journal impact

**Figure 4.**
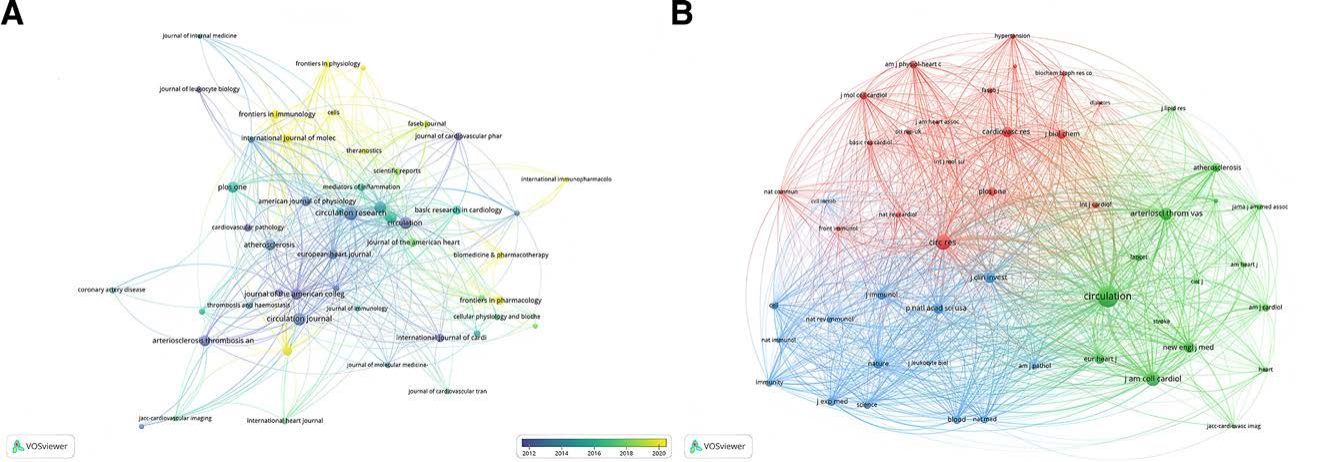
Visualization of journals (A) and co-cited journals (B) on the research of macrophages in myocardial infarction.

In this study, a comprehensive analysis was conducted on a corpus of 1342 articles published in 521 distinct journals. Table 2 provides a detailed enumeration of the top 10 journals, categorized by the volume of publications, along with their respective 2022 impact factors. Notably, 7 of these leading journals were positioned within the first quartile (Q1) of the Journal Citation Reports (JCR), indicating a high standard-of-citation influence within their respective fields. The geographical distribution of these publishers is also of interest, with 7 being headquartered in the United States, 1 in the United Kingdom, 1 in Japan, and 1 in Ireland. Among the top 10 journals, circulation emerged as the most influential, with an impact factor of 39.92, closely followed by the “European Heart Journal”, with an impact factor of 35.85. Additionally, 30% of the journals analyzed were classified within the Q1 category, underscoring the overall quality and impact of the publications within the scope of this study. Subsequently, a focused examination was performed on 50 journals, selected based on a minimum threshold of 5 relevant publications, to delineate the journal network, as shown in Figure 4A. This analysis revealed that circulation demonstrated robust citation interactivity with other notable journals, such as the “Journal of Molecular” and “Cellular Cardiology,” “Circulation’ Research,” and “Cardiovascular Research”.

**Table 2:** Top 10 journals and co-cited journals related to macrophages in myocardial infarction.

Rank	Journal	Count	IF	JCR	Co-cited journal	Citation	IF	JCR
1	Circulation Research	33 (2.459%)	20.1	Q1	Circulation	6088	39.8	Q1
2	Circulation Journal	28 (2.086%)	3.3	Q3	Circulation Research	3064	20.1	Q1
3	Circulation	27 (2.012%)	37.8	Q1	Journal of the American College of Cardiology	2639	24	Q1
4	Journal of Molecular and Cellular Cardiology	27 (2.012%)	5	Q2	Arteriosclerosis Thrombosis and Vascular Biology	2174	8.7	Q1
5	Cardiovascular Research	26 (1.937%)	10.8	Q1	New England Journal of Medicine	1689	158.5	Q1
6	Atherosclerosis	23 (1.714%)	5.3	Q1	Journal of Clinical Investigation	1688	15.9	Q1
7	Arteriosclerosis, Thrombosis, and Vascular Biology	22 (1.639%)	8.7	Q1	Nature	1368	64.8	Q1
8	Journal of the American College of Cardiology	22 (1.639%)	24	Q1	Cardiovascular Research	1486	10.8	Q1
9	PLOS ONE	22 (1.639%)	3.7	Q2	Proceedings of the National Academy of Sciences of the United States of America	1465	11.1	Q1
10	American journal of physiology. Heart and circulatory physiology	18 (1.341%)	4.8	Q1	European Heart Journal	1319	39.3	Q1

#### 3.4.2. Result of co-cited academic journals

Co-citation analysis serves as a quantitative metric to gauge the associative strength between scholarly articles, with the frequency of co-citations serving as an indicator of a journal’s scholarly impact within a specific research domain. Utilizing the VOSviewer software for visual analytics, a co-citation network was constructed for 50 journals that filtered out journals with a minimum co-citation of 200, facilitating insightful network visualization, as depicted in Figure 4B. Table 2 presents the top 10 co-cited journals, among which the leading 3 journals were amassed in excess of 10,000 co-citations each. ‘Circulation’ emerged as a preeminent journal with 6088 co-citations, followed by “Circulation Research” with 3064 co-citations, and the “Journal of the American College of Cardiology” with 2639 co-citations. Subsequent rankings included “Arteriosclerosis Thrombosis and Vascular Biology” with 2174 co-citations, and the “New England Journal of Medicine” with 1689 co-citations. Notably, all listed journals are ranked within the Q1 of the JCR. The distinguished position of “Circulation” as the most co-cited journal underscores its pivotal influence in MI macrophage research.

### 3.5. Research hotspot analysis

#### 3.5.1. Results of citation bursts

Figure S4, Supplemental Digital Content, https://links.lww.com/MD/P271 exhibits the top 25 most-cited references in the field, with “bursts” signifying publications that have received a notably higher citation count than the norm over a span of at least 2 years. The blue line signifies the observation period from 2003 to 2022, and the red line highlights the burst duration. Of these, 13 articles have concluded their citation bursts, while 7 are currently experiencing them. The publication with the highest burst value investigates the therapeutic efficacy of Canakinumab (a monoclonal antibody) in post-MI patients^[10]^; another significant work includes a meta-analysis on the diversity of aortic leukocytes in atherosclerosis.^[11]^ A focused study examines macrophage polarization towards a reparative phenotype to facilitate healing after MI,^[12]^ and another discusses inflammation following acute MI (AMI).^[13]^ Additionally, a study delves into cellular immunology and the steady state,^[14]^ while 3 others concentrate on the role of resident cardiac macrophages in myocardial injury.^[15–17]^

#### 3.5.2. Frequency and clustering results of keywords

**Figure 5.**
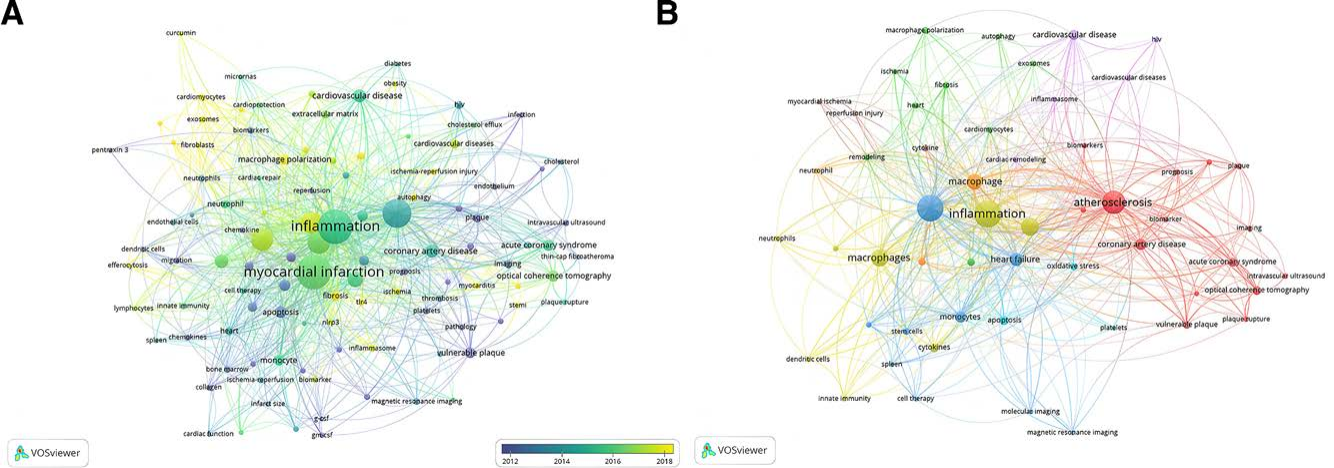
Keyword co-occurrence network (A). Time-overlapping co-occurrence analysis network of keywords (B).

In an analysis of 2341 keywords, 55 met the threshold of 10 occurrences and were further examined. Keywords similar to “MI” and “macrophage” were excluded from the search criteria. If the remaining keywords bore similar meanings, they were amalgamated. Notably, “inflammation” and “atherosclerosis” appeared over 200 times, underscoring the primary research focus on MI macrophages. Figure 5 illustrates the network visualization of these keywords. The frequency of keywords and their prominence are reflected in the node size, while the strength of their relationships is indicated by the proximity and thickness of the connecting lines. To encapsulate the key themes in new MI macrophage research, the 55 keywords were categorized into 8 groups. Closely related keywords were clustered together. Group 1, depicted in red, emphasizes keywords associated with atherosclerosis and early diagnosis and prevention, such as “atherosclerosis,” “acute coronary syndrome,” “biomarker,” and “prognosis.”

Group 2, represented in green, focuses on nouns related to various post-infarction manifestations and mechanisms, with keywords like “fibrosis,” “remodeling,” “angiogenesis,” “exosomes,” “autophagy,” and “macrophage polarization.” This clustering of terminology not only reflects a concentrated scholarly focus but also indicates a potential paradigm shift in the understanding of cardiac repair processes. The integration of these concepts points towards a more holistic approach to myocardial recovery, where the interplay between cellular regeneration, inflammatory response, and tissue adaptation is given paramount importance. As research delves deeper into the molecular intricacies of heart healing, the role of macrophages emerges as a pivotal factor, with their ability to modulate the cardiac environment post-infarction becoming increasingly evident. The exploration of macrophage polarization, in particular, offers promising insights into therapeutic strategies that could revolutionize the management of heart disease.

Group 3, colored in blue, includes keywords such as “stem cells,” “magnetic resonance imaging,” “molecular imaging,” and “monocytes,” along with immune organs “bone marrow” and “spleen.” This group highlights imaging terms related to MI and stem cell therapy, suggesting the potential of MRI in stem cell therapy for cardiac restoration (Rickers et al, 2004). It is noteworthy that the average publication year of research on stem cell therapy for cardiac restoration is quite early, implying that this field has not recently emerged in conjunction with MI macrophages. This might suggest a limited cross-disciplinary intersection between the 2 fields in recent times. Additionally, the clustering includes terms related to monocytes and various immune organs, with studies indicating that post-AMI, monocytes are recruited from various immune organs to the injured myocardium (Dutta & Nahrendorf, 2015), highlighting the significant role of monocytes in MI.

Group 4, in yellow, focuses on terms related to the inflammatory response mechanisms and inflammatory factors following AMI, mainly involving “inflammation,” “macrophages,” “innate immunity,” “dendritic cells,” and “neutrophils.”

Group 5, in purple, includes “cardiac remodeling,” “cardiovascular disease,” “HIV,” and “inflammasome.”

Group 6, in light blue, includes “Apoptosis,” “oxidative stress,” and “platelets.”

#### 3.5.3. Trend topic results of keywords

**Figure 6.**
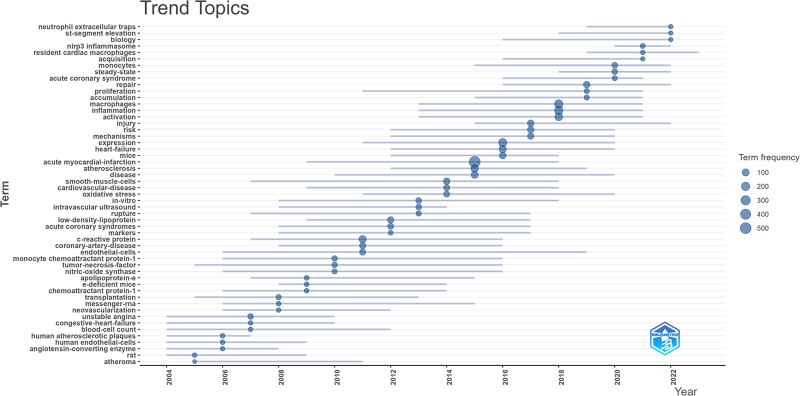
Trend topic analysis of researches about macrophages in myocardial infarction.

Trend topic analysis, as depicted in Figure 6, reveals that from 2003 to 2016, research predominantly concentrated on the fundamental mechanisms of MI, such as “necrosis” and “apoptosis.” Concurrently, themes like “acute coronary syndrome,” “atherosclerosis,” and “cardiovascular disease” indicate a broad scholarly focus on coronary diseases within the MI domain. These studies highlight the risk factors and the basic role of macrophages in coronary diseases.

The figure also references “intravascular ultrasound imaging” and “low-density lipoprotein,” likely reflecting technological advancements in medical imaging and biomarkers for the diagnosis of cardiovascular diseases.

Post-2016 trend topics, such as “resident cardiac macrophages,” “neutrophil extracellular traps,” “macrophages,” “monocytes inflammation,” “steady-state,” and “NLRP3 inflammasome,” suggest a research shift toward specific inflammatory mechanisms and immune cells, particularly the role of macrophages in the post-MI repair process. This period’s research unveils the diverse functions of macrophages in myocardial repair and regeneration, including their key roles in modulating inflammatory responses, promoting angiogenesis, and tissue remodeling.

The emergence of terms like “resident cardiac macrophages” and “neutrophil extracellular traps” signals an increased focus on macrophage subtypes and immune cells’ roles in MI in recent years.

#### 3.5.4. Bursts results of keywords

**Figure 7.**
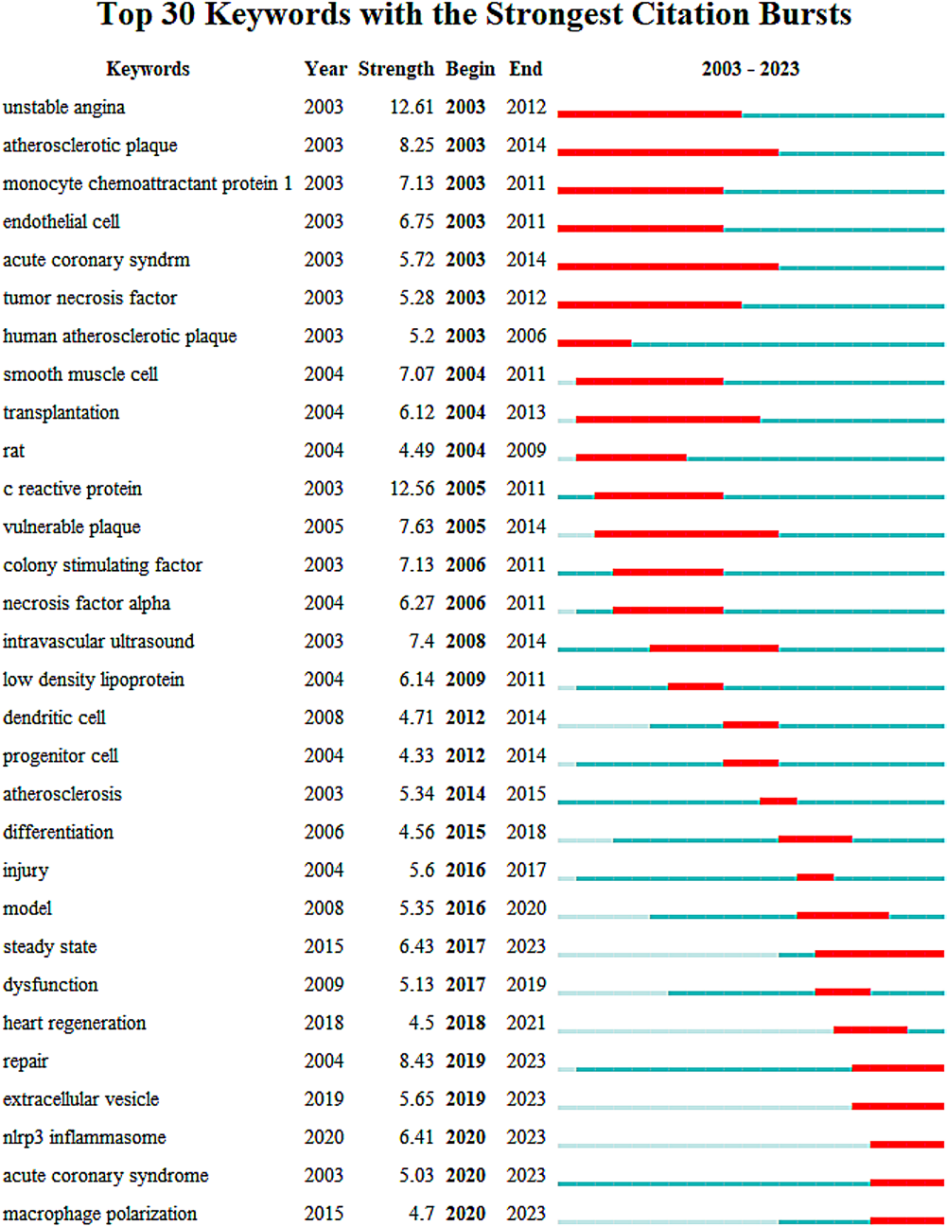
Top 30 Keywords with the strongest citation bursts.

Figure 7 enumerates the top 30 keywords that have exhibited the strongest citation bursts, persisting for at least 1 year. The keyword “unstable angina” (2005–2012) with a burst strength of 11.01 received the most significant attention. However, the continued citation burst of keywords such as “steady state” (2017–2023), “repair” (2019–2023), “extracellular vesicle” (2019–2023), “NLRP3 inflammasome” (2020–2023), “acute coronary syndrome” (2020–2023), “macrophage polarization” (2018–2023), and “macrophage” (2019–2023) indicates that these topics are likely to be the focal point of future research endeavors.

## 4. Discussion

### 4.1. Discussion of overview

Utilizing bibliometric methodologies, this study analyzed the growth patterns of MI in macrophage-related research from 2003 to 2023. The annual publication volume increased from an initial 35 to 102 papers per year, indicating sustained growth in this thematic literature. Since 2016, a surge in publications on MI macrophages has been observed, escalating from 67 annual publications in 2016 to 102 in 2019, with the field maintaining this level thereafter. This surge signifies a rapid development phase in MI macrophage research. The potential catalyst for this growth could be the burgeoning recognition of the role of macrophages in MI,^[18]^ sparking interest in their therapeutic potential.

Consequently, research institutions have significantly increased their support for studies on macrophages in MI, with increasing research funding propelling rapid development during this period.

The top 10 contributing countries published 1141 articles, accounting for 85.02% of the total, with potential overlaps due to transnational co-authorship. Among these nations, the United States and China dominate publication numbers. Moreover, international collaborations centered around the United States far exceed those of other countries. These findings affirm the significant contributions of the United States and leadership in MI macrophage research, likely stemming from the nation’s economic status and high healthcare investment levels. The field stands to benefit from extensive international collaboration, which enhances the overall caliber of research.

Eleven of the top 20 institutions are located in the United States, mirroring the countrywise distribution of publications. Despite China ranking second in publication numbers, only 4 institutions ranked it in the top 20, with their publication counts at the lower end. Although the Netherlands ranks third in publication numbers, 1 Dutch institution ranks fifth, with 30 papers.

Core journals frequently serve as conduits for disseminating pivotal research in specific academic domains. The volume and caliber of publications not only reflect the journal’s impact and relevance, but also provide strategic indicators for researchers in selecting appropriate platforms for manuscript submission. Circulation Research led to publication volume, with a total of 33 articles. The journal with the highest impact factor is Circulation (IF 37.8), followed by Circulation Research (IF 20.1). Impact factors and JCR are commonly used metrics to assess a journal’s influence, with the JCR categorizing all journals into 4 quartiles (Q1–Q4) based on impact factors. Among the top ten journals with the highest number of published articles, Q1 journals represent 70%. Moreover, despite China’s significant contributions to MI macrophage research, Asian publishers are underrepresented in the top ten journals. There is a need to establish and develop journals in Asia that have international influence.

This study seeks to identify and analyze the research hotspots that have been the focus of extensive investigation by scholars during a specific timeframe.Citation counts serve as one of the indicators of a publication academic influence.^[19]^ Highly cited publications often represent fundamental themes in a research field. Research hotspots can be identified by calculating citation counts and recognizing highly cited publications. In this study, the 10 most frequently cited publications, published between 2005 and 2020, primarily focused on the mechanisms of macrophages in coronary artery diseases, with a recent hotspot research shifting focus.

In 2006, Dr Erling Falk from Aarhus, Denmark authored a paper titled “Pathogenesis of Atherosclerosis,” discussing the development of atherosclerosis, a chronic inflammatory disease of medium and large arteries driven by lipids. The main focus was understanding why atherosclerosis, after years of indolent growth, suddenly complicates luminal thrombosis, leading to devastating consequences such as heart attacks and strokes. This study underscores the importance of detecting plaques prone to thrombosis to prevent such complications. Key points include the role of atherosclerotic stimuli; protective factors such as high-density lipoprotein (HDL) and exercise; susceptibility factors; and cellular components involved in atherosclerosis, such as endothelial cells, leukocytes, and smooth muscle cells^[20]^

In 2014, a paper published in The New England Journal of Medicine titled “HDL Cholesterol Efflux Capacity and Incident Cardiovascular Events” explored the role of HDL cholesterol efflux capacity and the ability of HDL to accept cholesterol from macrophagesin atherosclerotic cardiovascular diseases. This study investigated the association between cholesterol efflux capacity and cardiovascular events in a multi-ethnic cohort and found a negative correlation between efflux capacity and the incidence of cardiovascular events.^[21]^

In 2014, Lavine et al identified 4 cardiac macrophage populations, with Ly6c(hi) monocytes contributing to all 4 populations during macrophage depletion or cardiac inflammation.^[22]^.

In 2020, Guzik et al studied the complex interactions between COVID-19 and the cardiovascular system and provided insights into the mechanisms of cardiac injury and potential therapeutic strategies.^[23]^ The study mentioned that COVID-19 infection could lead to macrophage overactivation, resulting in a cytokine storm. Additionally, SARS-CoV-2 infection may cause macrophage and T-cell infiltration in the infected myocardium, leading to acute myocarditis and severe cardiac injury. The main findings of this study emphasize the necessity of early assessment and monitoring of cardiac injury and coagulation markers in COVID-19 patients, the role of ACE2 in viral invasion, and the potential benefits of renin–angiotensin–aldosterone system inhibitors.

### 4.2. Discussion of keywords co-occurrence analysis

Because keywords reflect the core content of a study, co-occurrence analysis can identify high-frequency keywords appearing across different studies, thus aiding researchers in quickly identifying research hotspots. In this study, the most common keywords were “Inflammation” and ‘atherosclerosis,’ appearing more than 200 times. Oxidative stress is another frequently occurring keyword. Oxidative stress is characterized by an imbalance between the production of free radicals and the body’s ability to counteract or detoxify their harmful effects using antioxidants.^[24]^ Studies have shown that in AMI, particularly post-reperfusion, a significant amount of reactive oxygen species (ROS) is generated in the ischemic myocardium.^[25]^ ROS directly damages cell membranes, leading to cell death. Furthermore, ROS stimulate signal transduction, producing inflammatory cytokines, such as tumor necrosis factor-alpha and interleukin (IL)-1β. ROS and inflammatory cytokines activate matrix metalloproteinases, which are enzymes that degrade the extracellular matrix,^[26]^ leading to myocardial fiber slippage and ventricular dilation and remodeling.^[27]^ After intense oxidative stress, activation of the mitochondrial quality control system is necessary to eliminate damaged components.^[28]^ Therefore, the importance of oxidative stress in MI macrophage research lies not only in its direct damaging effects on cells, but also in its critical role in ventricular remodeling, inflammatory response, and intercellular communication. Further investigation in this area may be an important target for future therapeutic strategies.

### 4.3. Discussion of burst detection

The “burst detection” method of CiteSpace can identify keywords or cited references that have undergone significant changes over time. Researchers can utilize keywords and cited references with burst features to explore emerging hotspots in their fields of study.^[29]^ In this research, the terms “repair” (2017–2023), “steady state” (2017–2023), “macrophage polarization” (2018–2023), “macrophage” (2019–2023), “extracellular vesicle” (2019–2023), “nlrp3 inflammasome” (2020–2023), and “acute coronary syndrome” (2019–2023) have all exhibited sustained bursts up to the year 2023.

NOD-like receptor family, pyrin domain containing 3 (NLRP3), as a star target in the field of macrophage mechanism research, is pivotal because of its central role in inflammatory responses and direct relevance to various diseases. The NLRP3 inflammasome is a multiprotein cytoplasmic complex belonging to the NLR protein family. It is primarily expressed in macrophages and detects products of damaged cells, such as extracellular ATP and crystalline uric acid, as components of inflammasomes.^[30]^ Upon NLRP3 assembly in response to cellular disturbances, activated caspase-1 is generated, promoting the maturation and release of inflammatory cytokines IL-1β and IL-18, as well as pyroptosis.^[31]^ Current research indicates that in patients with ST-segment elevation MI, activation of the NLRP3 inflammasome is closely associated with the amplification of inflammatory responses, tissue damage, and subsequent adverse cardiac remodeling.^[32]^ Inhibitors of the NLRP3 inflammasome not only show therapeutic effects in animal models, but drugs such as colchicine also demonstrate potential in clinical trials to reduce post-MI cardiac events. The COLCOT trial (NCT02551094) demonstrated that colchicine, an NLRP3 inflammasome inhibitor, reduced major cardiovascular events by 23% in post-MI patients. These developments suggest that macrophage-targeted therapies may soon complement current standard-of-care involving β-blockers and statins. Future research on MI will focus on a deeper understanding of the role of the NLRP3 inflammasome in different cardiovascular diseases and the development of more specific and effective NLRP3-targeted therapeutic strategies.^[33]^

The identification of burst keywords not only directs the trajectory of forthcoming research endeavors, but also the correlation and amalgamation among these burst terms may furnish scholars with innovative investigative paradigms. For instance, the interplay between macrophage polarization and exosomal dynamics is pivotal in orchestrating the reparative mechanisms of MI.

Macrophage polarization is a dynamic and intricate process by which mature macrophages differentiate into distinct functional phenotypes based on specific environmental cues. This polarization is crucial for pathogen defense, inflammation modulation, tissue repair, and the maintenance of homeostatic balance within biological systems.^[34]^ Extracellular vesicles (EVs), once considered as cellular debris, have become a focal point of scientific interest because of their complex cargo of proteins, RNA (predominantly non-coding), and lipids. Their role in cell communication is increasingly recognized, with the capacity to carry a multitude of biomolecules, thus serving as a key link in intercellular signaling. Exosomes, a subset of EVs, are particularly intriguing; usually measuring between 30 to 150 nanometers in diameter, they are generated through the endocytic pathway, where multivesicular bodies merge with the plasma membrane, expelling their contents into the extracellular milieu. Given their distinct biological traits and functionalities, exosomes are currently at the forefront of research in the field of extracellular vesicles.^[35,36]^

The cardiac environment is composed of a variety of cell types, including cardiomyocytes, endothelial cells, fibroblasts, smooth muscle cells, and macrophages, which coordinate to produce contractile myocardium.^[37]^ Following MI, these cellular communities secrete a range of paracrine factors involved in stress responses.^[38]^ Electron microscopy and immunohistochemistry have revealed that exosomes are vital conduits within the cardiac paracrine network.^[39]^

Traditionally, macrophages have been categorized into 2 main phenotypes: M1, associated with pro-inflammatory activities, and M2, related to reparative functions. These phenotypes are distinguished by their roles in the inflammatory response and tissue repair and are characterized by unique signaling pathways and cytokine release.^[40]^

**Figure 8.**
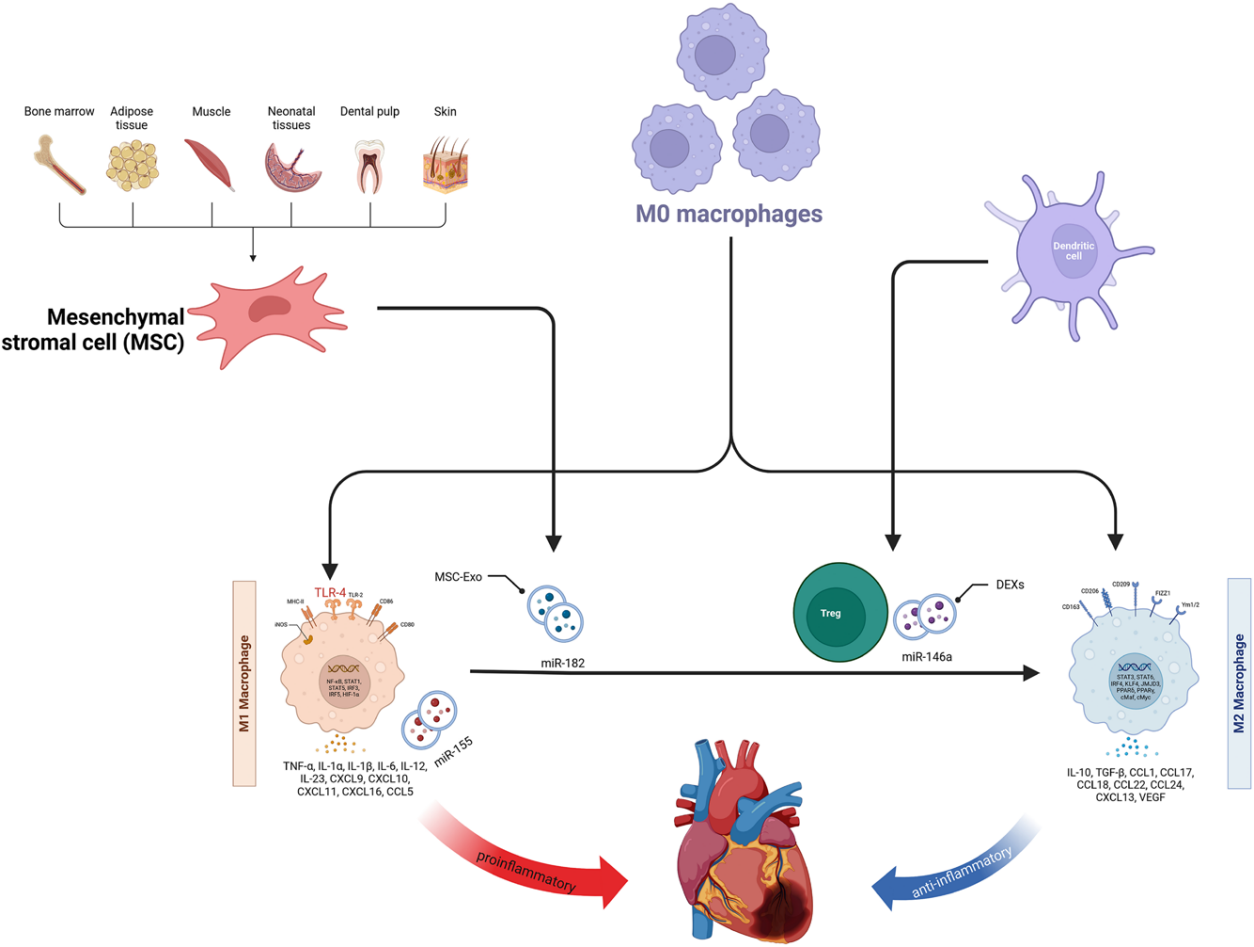
A mechanistic diagram illustrates the effect of extracellular vesicles on macrophage polarization in the context of myocardial infarction.

As shown in Figure 8, EVs originating from diverse cellular sources modulate macrophage polarization in the context of MI. These EVs foster a transition towards the M2 phenotype, thereby enhancing tissue repair and mitigating inflammatory responses.

Mesenchymal stem cells (MSCs) are a key cellular entity within the human body, and their unique immunomodulatory properties and propensity to promote tissue repair have garnered scientific attention. MSCs are adult stem cells known for their self-renewal and multilineage differentiation capabilities. They were initially discovered in the bone marrow but were later identified in a variety of tissues, including adipose tissue, umbilical cord blood, skeletal muscle, umbilical cord, and placenta. In the realm of MI, uncovering the beneficial effects of MSCs and their exosomes, as well as enhancing their therapeutic efficacy, presents a crucial research question.^[41,42]^ The work of Cho et al has shed light on the regulatory impact of MSCs on the immune environment, particularly in modulating the M1/M2 balance of bone marrow-derived macrophages (BMDMs). Thus, promoting cardiac repair MSCs with BMDMs significantly reduced the expression of M1 markers such as IL-6, IL-1β, MCP-1, and iNOS, while enhancing M2 markers such as IL-10, IL-4, CD206, and Arg1. The significant decrease in the iNOS to Arg1 ratio in BMDMs co-cultured with MSCs indicates that MSCs may catalyze cardiac repair by pushing the macrophage phenotype towards M2.^[43]^ Additionally, Zhao et al uncovered the pivotal role of MSC-derived exosomes (MSC-Exos) in ameliorating myocardial ischemia–reperfusion injury. MSC-Exos have been shown to coordinate macrophage polarization by transporting miR-182, thereby mitigating myocardial ischemia–reperfusion injury. Both in vitro and in vivo experiments confirmed that MSC-Exos reduced the M1 macrophage population while enhancing the generation of M2 macrophages. In MSC-Exos, miR-182 has been identified as a key regulator of macrophage polarization by targeting toll-like receptor 4.^[44]^

In addition to MSCs, dendritic cell (DC)-derived exosomes (DEXs) also play an essential role in macrophage polarization. DCs are antigen-presenting cells, and their secreted DEXs have a significant impact on modulating immune responses and promoting tissue repair. Post-MI, signals released from cardiac tissue can activate DCs to secrete DEXs.^[45,46]^ For instance, Choo et al found that DCs stimulated with MI tissue lysates or serum from MI mice activated Treg cells and induced a shift of macrophages from the M1 to the M2 phenotype, thereby improving cardiac repair and function. Moreover, specific miRNAs in DEXs, such as miR-146a, have been shown to negatively regulate inflammatory responses by inhibiting key pro-inflammatory signaling pathways such as nuclear factor kappa-light-chain-enhancer of activated B cells, reducing the activation of M1 macrophages, and promoting the polarization of M2 macrophages.^[47]^

Similarly, in the pathological process of MI, the direct effects of macrophage-derived exosomes on the cardiac cells should not be overlooked. M1 macrophages release exosomes carrying a plethora of pro-inflammatory miRNAs such as miR-155. miR-155 can be taken up by cardiac endothelial cells and fibroblasts, inhibiting angiogenesis and exacerbating cardiac dysfunction in endothelial cells by targeting a range of genes, including Rac family small GTPase 1 (RAC1), p21-activated kinase 2 (PAK2), deacetylase sirtuin 1 (Sirt1), and protein kinase AMP-activated catalytic subunit alpha 2 (AMPKα2).^[48]^ Furthermore, in fibroblasts, miR-155 inhibits proliferation by reducing the expression of a key mediator of FB proliferation, Son of Sevenless 1 (Sos1), and simultaneously downregulating the expression of cytokine signaling inhibitor 1 (SOCS1), further promoting the inflammatory response.^[49]^

In summary, the unparalleled therapeutic potential of exosomes in the treatment of MI macrophages is evident. As research delves deeper, we can anticipate the development of novel therapeutic strategies aimed at promoting cardiac tissue repair, reducing inflammation, and enhancing cardiac function.

However, much of the current research is based on the traditional M1–M2 framework of macrophages, which is a recent immunological challenge. Studies have shown that after MI, cardiac monocytes and macrophages form a heterogeneous ensemble, exhibiting diverse origins, dynamics, and functions.^[50]^ This heterogeneity transcends the conventional M1/M2 dichotomy. Using single-cell RNA sequencing (scRNA-seq), researchers have delineated multiple macrophage subpopulations within cardiac tissue. The authors identified 6 distinct macrophage and dendritic cell subpopulations in control mice using t-distributed stochastic neighbor embedding analysis. In contrast, 13 different macrophage subpopulations were identified in MI mice, 7 of which were unique to post-MI conditions.^[16]^ Moreover, when macrophages are exposed to a broader range of cytokines, they exhibit a spectrum of phenotypes, rather than a binary shift from M1 to M2. It has been observed that macrophages can simultaneously express characteristic markers of both M1 and M2 phenotypes, and can even adopt markers of other subpopulations in vitro.^[51]^ This evidence suggests that while the M1–M2 paradigm is useful, it represents an oversimplification of macrophage polarization.

The current understanding necessitates a reevaluation of the M1–M2 framework, especially in the context of in vivo experiments. Targeting specific transcriptional pathways may affect only certain subpopulations within the M1 or M2 categories, highlighting the limitations of the existing paradigm in accurately defining macrophage subpopulations.

The complex interplay between macrophages and their microenvironment underscores the need for a more refined classification system. The remarkable plasticity of macrophages is orchestrated by a complex network of transcription factors, epigenetic modifiers, and post-transcriptional regulators that respond to various local and systemic signals. This adaptive capacity allows macrophages to play multifaceted roles in both health and disease. For future research, it is crucial to dissect the full spectrum of macrophage phenotypes and clarify their individual and collective contributions to physiological and pathological states. Such endeavors will not only deepen our understanding of macrophage biology, but may also reveal new therapeutic targets within the immune system. Pursuing this knowledge will help elucidate the nuanced roles of macrophages beyond traditional paradigms, ushering in a new era of immunological discovery.

In 2023, 7 cited references will continue to exhibit significant citation bursts. Notably, the article “Antiinflammatory Therapy with Canakinumab for Atherosclerotic Disease” achieved the highest citation burst value from 2003 to 2023 (citation burst value = 18.13) and is currently experiencing a citation surge. Published in the New England Journal of Medicine in 2017, this pivotal study evaluated the efficacy of canakinumab, a monoclonal antibody, in patients with post-MI through a randomized, double-blind trial with 10,061 participants.^[45]^ This multicenter RCT (n = 10,061) demonstrated that subcutaneous 150 mg canakinumab significantly reduced recurrent cardiovascular events (HR 0.85, 95% CI 0.74–0.98) independent of lipid-lowering effects. Mechanistically, IL-1β is predominantly secreted by activated macrophages through NLRP3 inflammasome signaling – a key pathway in macrophage-mediated sterile inflammation post-MI.^[30,52]^ In atherosclerotic plaques, macrophage-derived IL-1β drives endothelial activation via nuclear factor kappa-light-chain-enhancer of activated B cells, promoting monocyte recruitment and foam cell formation.^[53]^ The trial’s 34.2% reduction in hs-CRP (*P* < .001) specifically reflects attenuated macrophage-driven inflammation, as CRP production is directly stimulated by IL-1β through hepatic IL-6 induction.^[54]^ Notably, canakinumab’s selective IL-1β neutralization reshapes macrophage polarization by reducing M1 markers (CD86+; *P* < .01) while preserving reparative M2 populations (CD206+; *P* = .32) in human carotid plaques, explaining its efficacy in stabilizing high-risk coronary lesions.^[55]^

Furthermore, 3 studies focused on the role of resident cardiac macrophages in cardiac injury.^[15–17]^ Aligning with keyword trends and topic analysis outcomes.

Resident cardiac macrophages are increasingly being recognized as pivotal players in cardiovascular research, with bibliometric analyses underscoring their emerging prominence across multiple scientific dimensions. These cells, which exhibit long-term persistence and self-renewal within cardiac tissues, are integral to safeguarding cardiac function and mitigating adverse remodeling after MI.^[56]^ Fate mapping experiments have shed light on their origins, predominantly tracing back to the yolk sac.^[57]^ The research conducted by Dick et al revealed that while resident cardiac macrophages represent a minor proportion of the total macrophage population in the infarcted region, their absence significantly compromises cardiac function and hinders effective infarct healing.^[16]^ Complementary findings suggest that the depletion of CCR2 + tissue-resident macrophages in mouse models of dilated cardiomyopathy increases mortality rates and has detrimental effects on ventricular remodeling and coronary angiogenesis.^[58]^ Moreover, macrophages that are recruited to infarcted cardiac tissue from immune organs and the bloodstream post-MI demonstrate remarkable plasticity. These cells partially adopt transcriptional characteristics reminiscent of resident macrophages. Nonetheless, advanced techniques such as flow cytometry and scRNA-seq can discern recruited macrophages from the resident population, as they do not completely mirror the core transcriptional traits of resident cardiac macrophages.^[16]^

These insights are instrumental in unraveling the complex alterations in macrophage populations within cardiac tissues following an infarction and their pivotal role in the mechanisms underlying cardiovascular injury repair. The distinction between resident and recruited macrophages is not merely a matter of origin but also of function and potential impact on therapeutic outcomes.

An evolving understanding of macrophage heterogeneity in the heart calls for a redefined approach to cardiovascular therapy. Targeting specific macrophage populations could offer a more precise method for promoting cardiac repair and regeneration. Future research should focus on the functional characterization of these distinct macrophage subsets and their interactions with the cardiac microenvironment. This will not only enhance our understanding of cellular dynamics post-infarction but also facilitate the development of targeted interventions to improve patient prognosis following myocardial injury.

This study has several limitations. First, it includes only articles in English indexed in the WoSCC database. However, since WoSCC covers more than 90% of high-impact studies, this limitation did not significantly distort the observed trends. Second, citation-based metrics inherently favor early publications, which may undervalue recent high-impact studies due to citation lag effects. Third, bibliometrics cannot assess research quality; for example, highly cited articles may contain conclusions that have been refuted by subsequent studies.

## 5. Conclusions

In recent years, research on macrophages in MI has garnered increasing attention. The significant rise in annual publications underscores the growing importance of this research area. This study identifies the countries and institutions globally involved in MI macrophage research, with the United States contributing the most and Circulation Research being the most active journal. Despite inherent methodological limitations of bibliometric analysis, this study systematically maps the evolving landscape of MI macrophage research. The identified trends (particularly the emerging focus on NLRP3 inflammasome regulation and macrophage polarization dynamics, the spatiotemporal heterogeneity of cardiac macrophages, and the exosome crosstalk between macrophages and stromal cells) provide actionable insights for prioritizing translational research.

Firstly, macrophage polarization dynamics (especially NLRP3 inflammasome-driven M1 activation and therapeutic M2 polarization) have emerged as key regulators of post-infarction remodeling, with clinical trials demonstrating the efficacy of IL-1β inhibition. Secondly, emerging scRNA-seq and flow cytometry technologies have identified over 13 functionally distinct macrophage subpopulations. Thus, the spatiotemporal heterogeneity of cardiac macrophages necessitates precise targeting strategies to maintain myocardial homeostasis. For example, precision targeting to protect or replenish CCR2 + resident macrophages may become a novel therapeutic target. Thirdly, exosome crosstalk between macrophages and stromal cells (e.g., MSC-derived miR-182 and macrophage miR-155) has unveiled new diagnostic biomarkers and cell-free therapeutic carriers.

Future research directions should prioritize the development of higher-resolution and in-depth spatial transcriptomics platforms to map macrophage functional niches; standardization of extracellular vesicle production at the clinical level; and investigation of more precise targeting strategies. This study lays the foundational framework for transitioning from broad anti-inflammatory approaches to precise immune modulation in post-MI care.

## Author contributions

**Conceptualization:** Jia Lin Zhong, Hao Wang.

**Data curation:** Guo Yang, Jia Lin Zhong.

**Funding acquisition:** Jun Xiao.

**Methodology:** Chuan Wei Li, Hao Wang.

**Project administration:** Yang Xing.

**Software:** Guo Yang.

**Supervision:** Guo Yang, Yang Xing, Chuan Wei Li, Jun Xiao.

**Visualization:** Guo Yang.

**Writing – original draft:** Guo Yang, Jia Lin Zhong.

**Writing – review & editing:** Jun Xiao.

## Supplementary Material


