# Supplementary material for: Evolutionary patterns and research frontiers of macrophages in myocardial infarction: A bibliometric analysis
Source: Medicine (Baltimore). 2025 Jun 27;104(26):e43038. doi: 10.1097/MD.0000000000043038 (PMC12212758; doi:10.1097/MD.0000000000043038)
Supplement: Supplementary file 1 [file medi-104-e43038-s001.docx]

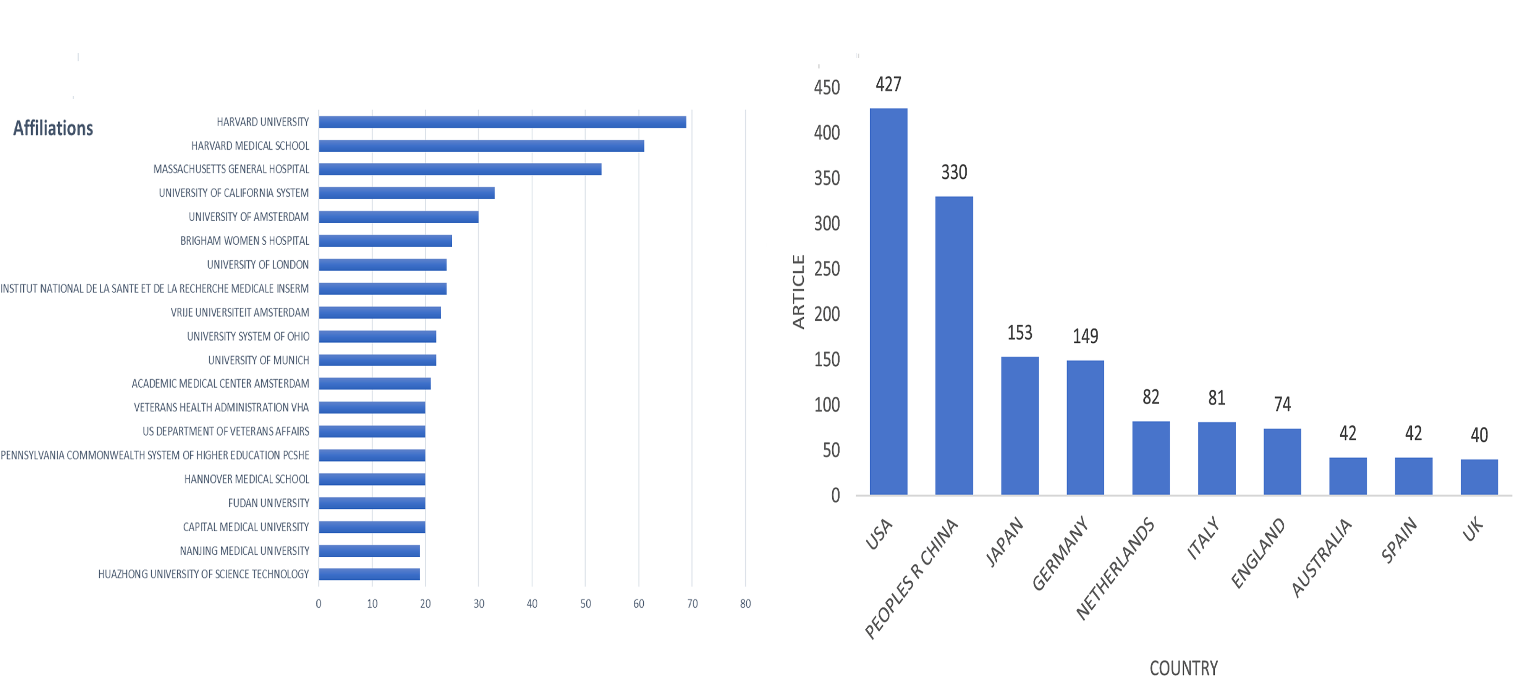


**Supplementary Figure 1**. The top 10 coutries and institutions with the most publications in the field of myocardial infarction macrophages.


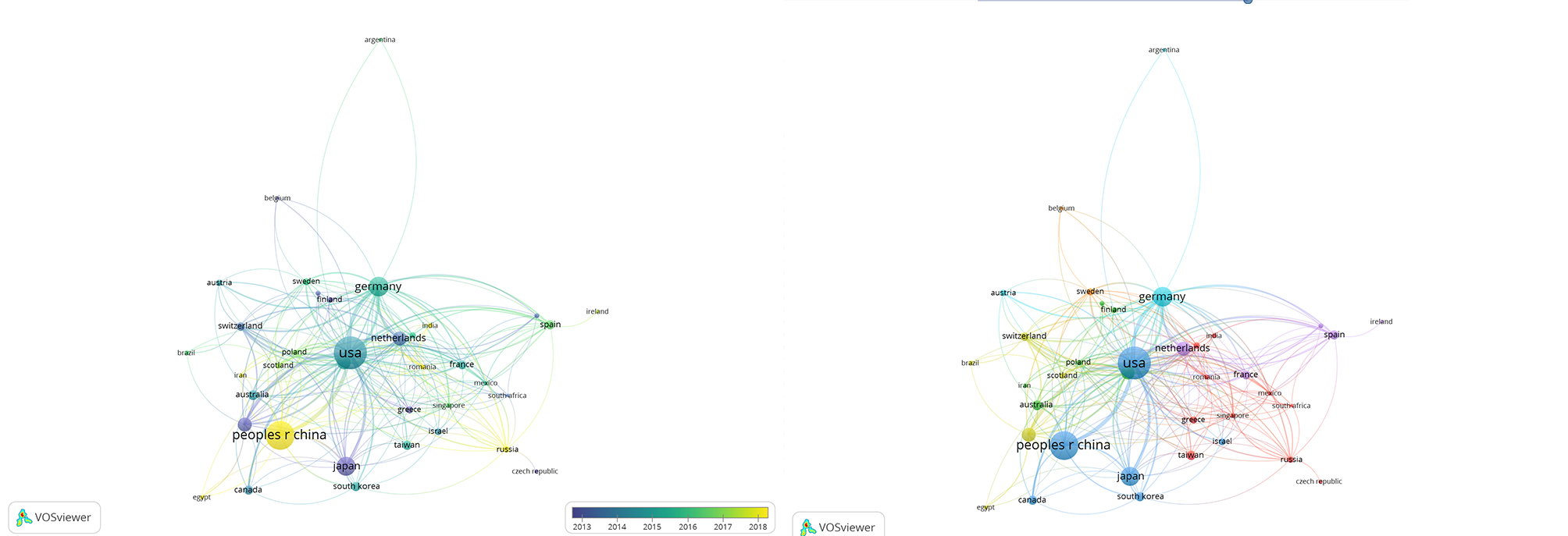


**Supplementary Figure 2**. Graphical Representation of Country Co-authorship Analysis, Network

Clustering and Time-Overlapping Network.


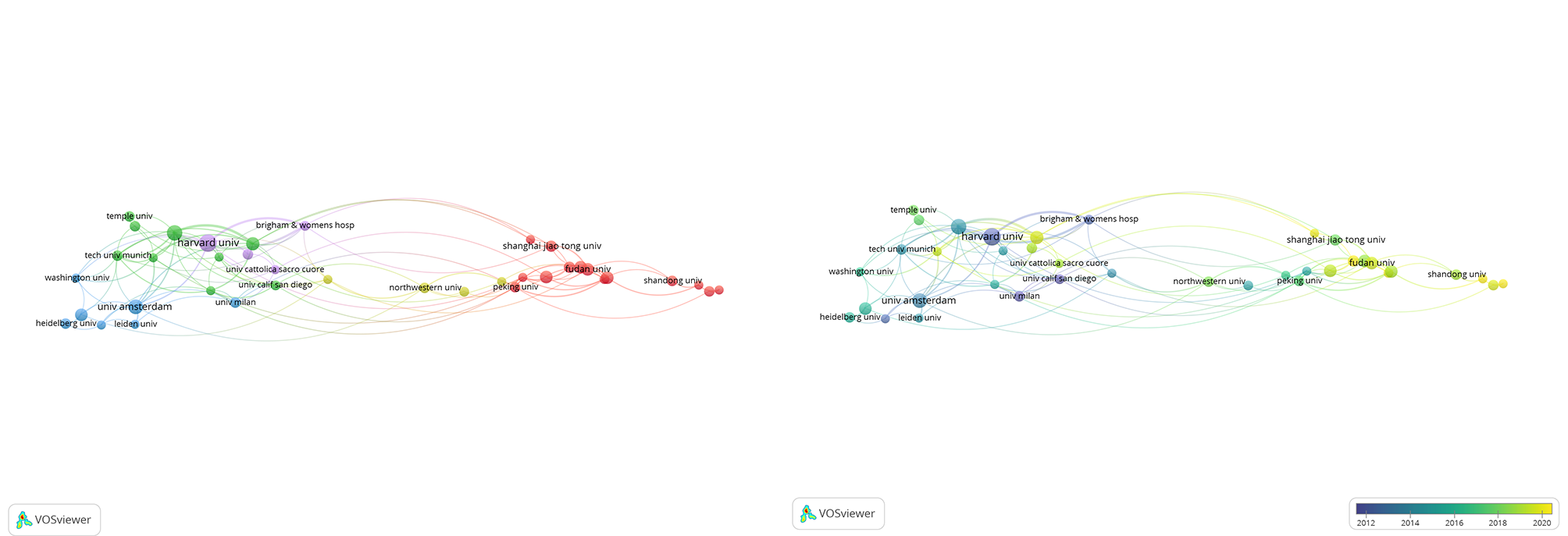
**Supplementary Figure** **3**. Graphical Representation of Institutional Co-authorship Analysis, Network Clustering and Time-Overlapping Network.
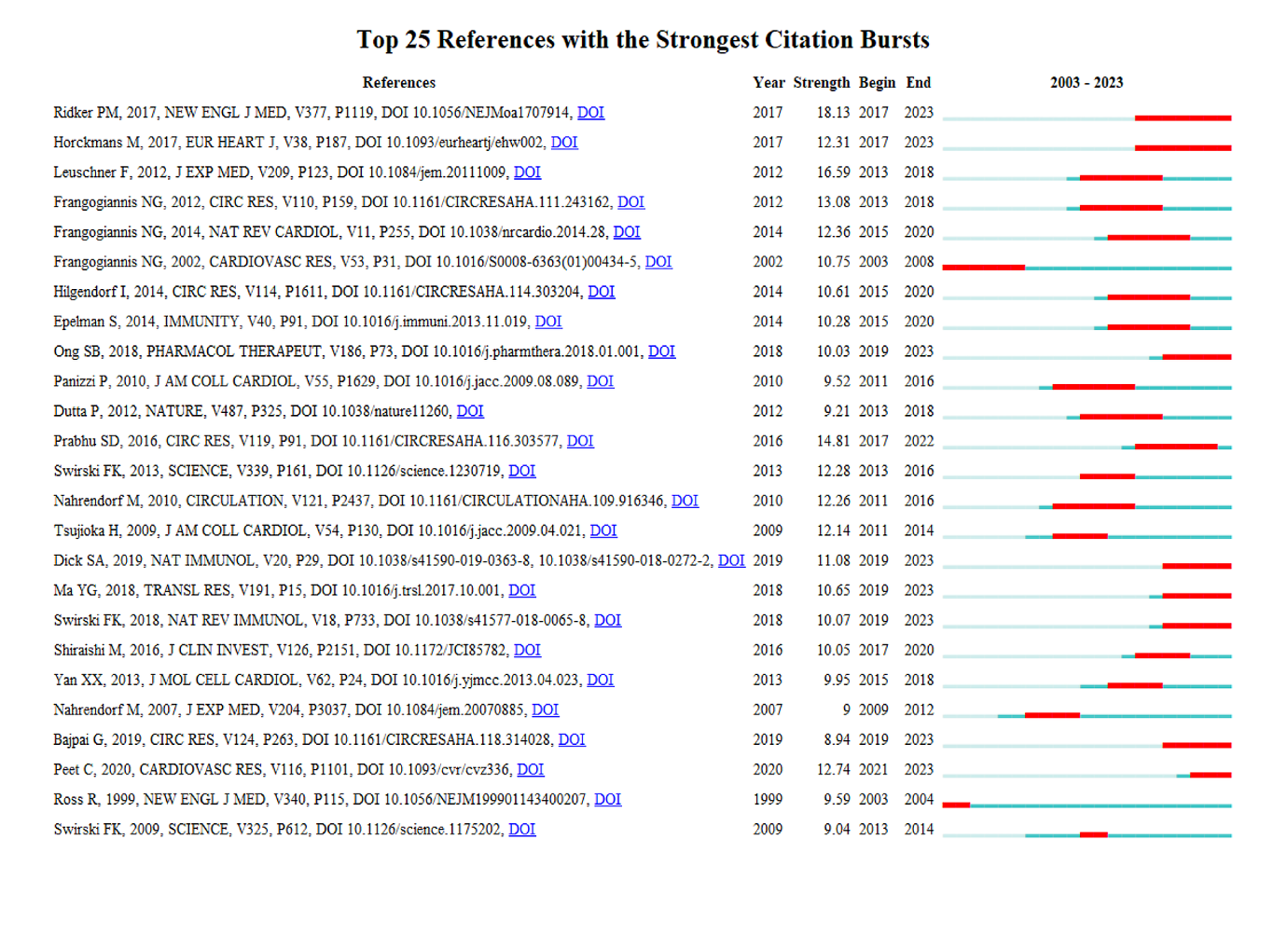


**Supplementary Figure 4**. Top 25 Most Frequently Cited References in the Field of macrophages in myocardial infarction.
